# Meta‐Device for Field‐of‐View Tunability via Adaptive Optical Spatial Differentiation

**DOI:** 10.1002/advs.202412794

**Published:** 2025-01-13

**Authors:** Yin Zhou, Lin Li, Junhao Zhang, Jialuo Cheng, Xiaoyuan Liu, Yunhui Gao, Zihan Geng, Lei Li, Junxiao Zhou, Mu Ku Chen

**Affiliations:** ^1^ Department of Electrical Engineering City University of Hong Kong Kowloon Hong Kong 999077 China; ^2^ School of Electronics and Information Engineering Sichuan University Chengdu 610065 China; ^3^ State Key Laboratory of Terahertz and Millimeter Waves City University of Hong Kong Kowloon Hong Kong 999077 China; ^4^ Institute of Data and Information Tsinghua Shenzhen International Graduate School Tsinghua University Shenzhen Guangdong 518071 China; ^5^ Centre for Biosystems, Neuroscience, and Nanotechnology City University of Hong Kong Kowloon Hong Kong 999077 China

**Keywords:** meta‐devices, optical computing, optical differentiation

## Abstract

Optical edge detection is a crucial optical analog computing method in fundamental artificial intelligence, machine vision, and image recognition, owing to its advantages of parallel processing, high computing speed, and low energy consumption. Field‐of‐view‐tunable edge detection is particularly significant for detecting a broader range of objects, enhancing both practicality and flexibility. In this work, a novel approach—adaptive optical spatial differentiation is proposed for field‐of‐view‐tunable edge detection. This method improves the ability to acquire spatial information and facilitates edge detection over a wider angular range. The adaptive optical spatial differentiation meta‐device relies on two core components: the spatial differentiation dielectric metasurface and the adaptive liquid prism. The meta‐device is shown to function as a highly efficient (≈85%) isotropic spatial differentiator, operating across the entire visible spectrum (400 to 700 nm) within a wide‐angle object space, expanding up to 4.5 times the original field of view. The proposed scheme presents new opportunities for efficient, flexible, high‐capacity integrated data processing and imaging devices. And simultaneously provides a novel optical analog computing architecture for the next generation of wide field‐of‐view phase contrast microscopy.

## Graphical abstract

A larger FOV enables edge identification over a broader area, crucial for detecting boundaries in complex or large‐scale images. To meet this need, an adaptive optical spatial differentiation meta‐device is developed. This device acts as a highly efficient (85%) isotropic spatial differentiator, operating across the visible spectrum in a wider‐angle object space, expanding up to 4.5 times the original FOV.

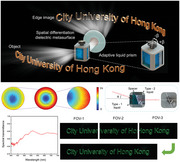

## 1 Introduction

The emergence of optical analog computing has paved the way for new avenues to extract image information swiftly and effectively.^[^ ^1^, ^2^, ^3^ ^]^ Edge detection is pivotal in fields like machine vision, biomedical imaging, and industrial inspection.^[^ ^4^ ^]^ Traditional edge detection heavily relies on computationally intensive digital processing, leading to time and energy inefficiencies.^[^ ^5^, ^6^ ^]^ With the advent of optical analog computing, characterized by high‐speed operations and low energy consumption, optical edge detection is more efficient and practical based on surface plasmonics,^[^ ^7^ ^]^ photonic crystals,^[^ ^8^, ^9^ ^]^ photonic spin Hall effect,^[^ ^10^, ^11^ ^]^ and multilayered structures.^[^ ^12^ ^]^

Metasurfaces, comprising subwavelength‐scale units engineered to manipulate light fields for diverse functionalities,^[^ ^13^ ^]^ such as multi‐function imaging,^[^ ^14^, ^15^, ^16^, ^17^, ^18^, ^19^, ^20^, ^21^, ^22^, ^23^, ^24^ ^]^ holographic display,^[^ ^25^, ^26^, ^27^, ^28^, ^29^, ^30^, ^31^ ^]^ nonlinear optical response,^[^ ^32^, ^33^, ^34^, ^35^ ^]^ multiphoton quantum source,^[^ ^36^ ^]^ and mathematical operations,^[^ ^37^, ^38^ ^]^ including optical spatial differentiation.^[^ ^39^, ^40^, ^41^, ^42^, ^43^, ^44^, ^45^, ^46^, ^47^ ^]^ Despite these advancements, achieving field‐of‐view‐tunable edge detection poses a significant challenge in current research. Field‐of‐view‐tunable edge detection is of paramount importance, facilitating edge detection of a greater array of objects and augmenting the acquisition capacity of object‐spatial information.

Considerable research efforts have been devoted to obtaining wide‐angle images through techniques such as image stitching,^[^ ^48^ ^]^ and zoom imaging.^[^ ^49^ ^]^ However, these methods are encumbered by bulky and complex structures and intensive operations. Another alternative approach, based on the traditional mechanical beam steering mechanisms like the Risley prism^[^ ^50^ ^]^ and galvanometer,^[^ ^51^ ^]^ typically suffers from complex structures. Although some novel beam steering devices have recently been proposed,^[^ ^52^, ^53^, ^54^, ^55^, ^56^ ^]^ they predominantly exhibit polarization dependency or wavelength selectivity as well as the requirement for algorithmic post‐processing. Therefore, these methods are difficult to apply directly for optical imaging applications. Adaptive photonic devices provide possibilities for solving these problems. In recent years, significant research efforts have been dedicated to exploring the potential of adaptive photonic devices in optical systems, owing to their compactness, versatility, and extended functionalities. Adaptive photonic devices offer a range of applications, including tunable focal length,^[^ ^57^, ^58^ ^]^ aberration correction,^[^ ^59^ ^]^ and optical steering.^[^ ^60^ ^]^ Adaptive photonic devices possess advantages such as lightweight, compactness, and tunability compared to conventional beam steering methods.

**Figure 1:**
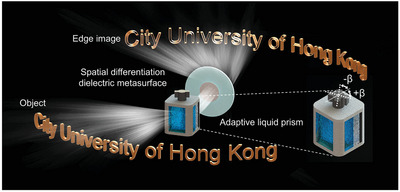
Working principle of the proposed adaptive optical spatial differentiation. The light incident onto different fields of view of the object passes through the proposed meta‐device based on the spatial differentiation dielectric metasurface and adaptive liquid prism (by rotating angle *β*), and finally, its completive edge information is obtained at the image plane.

In this study, we propose a novel adaptive optical spatial differentiation approach, the concept of field‐of‐view‐tunable edge detection. Our aim is to enhance the acquisition of object‐spatial information and edge detection for more objects within the object space, including amplitude objects and phase objects. The core components of the proposed adaptive optical spatial differentiation consist of a spatial differentiation dielectric metasurface and an adaptive photonic device (adaptive liquid prism). These components confer upon the meta‐device the capabilities of edge detection and field‐of‐view tunability, as shown schematically in **Figure** **1**. Through a synergistic integration of beam steering and edge detection mechanisms, the proposed meta‐device enables the acquisition of edge information in object space while dynamically adjusting the field of view. Moreover, this meta‐device exhibits isotropic spatial differentiation characteristics applicable across the entire visible spectrum (See Note S1, Supporting Information for comparing our adaptive optical spatial differentiation meta‐device with the main features of previous classic optical differentiation calculations based on metasurfaces). Our research contributes to the advancement of optical analog computing techniques, expanding the scope of research in this field, which provides a novel framework for the next generation of advanced phase contrast microscopy.

## 2 Results

### 2.1 Design and Structure of the Adaptive Optical Spatial Differentiation Meta‐Device

**Figure 2:**
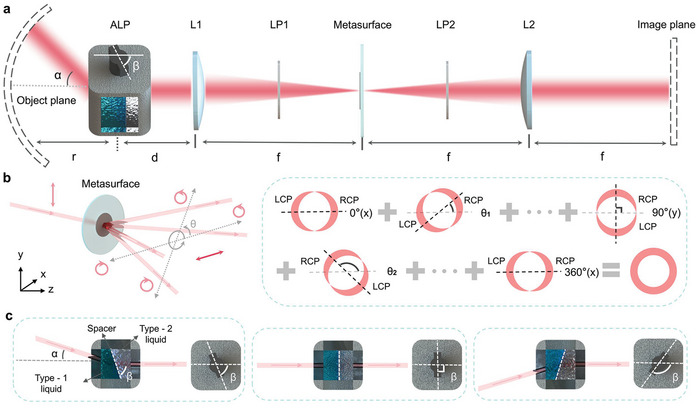
Principle of the adaptive optical spatial differentiation meta‐device. a) Schematic structure of the meta‐device. By rotating the shaft angle *β* of ALP, the incident light with the object information from the field angle *α* can be collected and then processed by the metasurface, the edge image can be captured at the image plane. ALP: adaptive liquid prism. L1 and L2: a pair of lenses to form a 4*f* system. LP1 and LP2: a pair of crossed linear polarizers. Proposed concept and detailed working principle of b) the spatial differentiation dielectric metasurface for edge detection and c) the adaptive liquid prism for field‐of‐view tunability.

The structure of the adaptive optical spatial differentiation meta‐device is shown in **Figure** **2**a. The significant components of the proposed meta‐device are the adaptive liquid prism and the spatial differentiation dielectric metasurface, which are able to achieve the functions of field‐of‐view (FOV) deflection and edge detection, respectively. Lens 1 and Lens 2 are combined to form a 4*f* system, and the spatial differentiation dielectric metasurface is placed at the Fourier plane. Two orthogonal‐linear polarizers are placed in the front and back of the spatial differentiation dielectric metasurface, which constitutes an optical spatial differentiator. The adaptive liquid prism is located at the front distance *d* of Lens 1, and its function is to deflect the FOV in the object space to detect objects with different field angles. The object is placed on the object plane at a distance *r* away from the adaptive liquid prism so that the proposed meta‐device can capture different angles of FOV and achieve wider‐angle edge detection. The relationship between *r* and *d* is *r* + *d* = *f_equ_* (*f_equ_* is the equivalent focal length of Lens 1), which can eliminate the aberration caused by object placement.

#### 2.1.1 Working Principle of the Spatial Differentiation Dielectric Metasurface

The working principle of the spatial differentiation dielectric metasurface is shown in Figure 2b, which is based on spin‐dependent splitting. Here, we illustrate and explain its principle of optical spatial differentiation from anisotropy to isotropic in detail. The proposed spatial differentiation dielectric metasurface is placed in the Fourier plane in the 4*f* system to be constituted as an optical spatial differentiator. In Figure 2b, when the *y*‐linear‐polarized plane wave incidents on the circle‐pattern object, the *y*‐linear‐polarized plane wave with information about the circle‐pattern object impacts the spatial differentiation dielectric metasurface. Two same images are created according to left‐handed circular polarization (LCP) and right‐handed circular polarization (RCP) after the linear polarization (LP) beam splitting because of the function of Pancharatnam‐Berry phase^[^ ^61^, ^62^ ^]^ gradient metasurface,^[^ ^63^ ^]^ which only contains a lateral (*x*‐axial) shift. After that, the overlapped light area by the LCP and the RCP is recombined into the LP light area, which the orthogonal‐linear polarizer will block. The edge information (the LCP and the RCP) remains on the CMOS camera in the end, as shown in the 0°(*x*) pattern of Figure 2b, which is anisotropic. It should be pointed out that the proposed spatial differentiation dielectric metasurface is isotropic, so the phase gradient direction is *θ* range from 0° to 360°. By combining the whole angles' anisotropic images, the final isotropic edge image is obtained. The formation process of the final isotropic edge image is shown on the right side of Figure 2b. A circle‐pattern object's final edge detection result is a uniform thickness ring. The designed phase of the spatial differentiation dielectric metasurface can be expressed as,

(1) $$\varphi \left(r,\theta \right)=\left[\exp\left(i\cdot \frac{2\pi }{\Lambda }\cdot r\right)\left(\begin{array}{cc} 1 & -i\\ i & 1 \end{array}\right)+\exp\left(-i\cdot \frac{2\pi }{\Lambda }\cdot r\right)\left(\begin{array}{cc} 1 & i\\ -i & 1 \end{array}\right)\right]$$

in which $\left(\begin{array}{cc} 1 & -i\\ i & 1 \end{array}\right)$ and $\left(\begin{array}{cc} 1 & i\\ -i & 1 \end{array}\right)$ represent the phase response to LCP and RCP components, respectively. The detailed mathematical expression of the whole working process of optical spatial differentiation is demonstrated as well (See Note S2, Supporting Information).

#### 2.1.2 Working Principle of Adaptive Liquid Prism

The working principle of the adaptive liquid prism for FOV tunability is shown in Figure 2c. There is a spacer in the adaptive liquid prism, and its function is to divide two types of liquid material (Type‐1 and Type‐2) with different refractive indexes into two cavities. When turning the spacer to different rotating angles *β*, the object information of different FOV angles *α* can be detected. Notably, the proposed adaptive liquid prism can achieve continuous lateral deflection of FOV. The correlation between the angle of FOV deflection *α* and the rotating shaft angle of the spacer *β* can be expressed as follows:

(2) $$\alpha =\arcsin\left(\cos\beta \sqrt{n_{2}^{2}-n_{1}^{2}\cos^{2}\beta }-n_{1}\sin\beta \cos\beta \right),\beta \subset \left(45^{\circ },135^{\circ }\right)$$

in which *n*_1_ and *n*_2_ correspond to the refractive indexes of Type‐1 liquid and Type‐2 liquid, respectively (See Note S5, Supporting Information for detailed mathematical derivation). The rotation range for the spacer *β* is contingent upon the structural dimensions of the adaptive liquid prism.

### 2.2 Fabrication and Characterization of the Spatial Differentiation Dielectric Metasurface and the Adaptive Liquid Prism

#### 2.2.1 Spatial Differentiation Dielectric Metasurface

**Figure 3:**
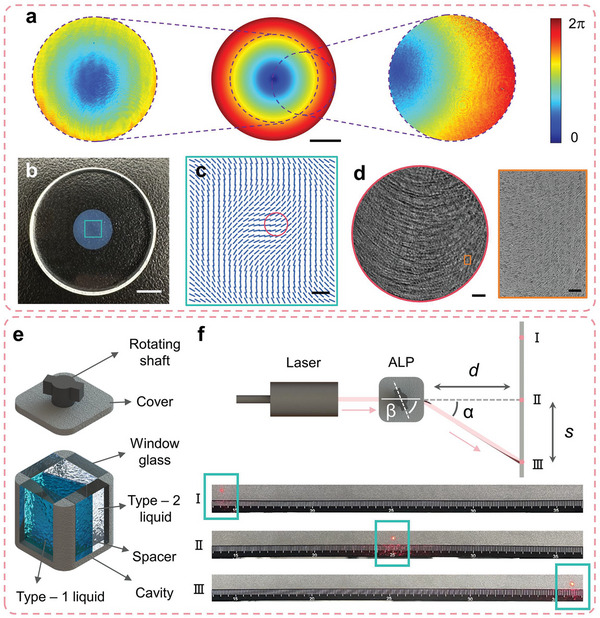
Characterizations of spatial differentiation dielectric metasurface and adaptive liquid prism. a) Designed and measured phases of the metasurface. Scale bar: 2 mm. b) Optical image of the metasurface. Scale bar: 5 mm. c) Orientation of the nanostructure in the green box of (b). Scale bar: 500 µm. d) Polariscope optical image shows the detailed structure of the red circle of (c). Scale bar: 50 µm. Right panel, top view of scanning electron microscope (SEM) of the orange box. Scale bar: 5 µm. e) Structures of the adaptive liquid prism. f) Experimental setup and results of deflection characterization of the adaptive liquid prism.

The designed and measured phases of the spatial differentiation dielectric metasurface are shown in **Figure** **3**a, and the measured phases are obtained by the commercial measurement system (AR‐Meta‐P, IDEAOPTICS INC). Figure 3b shows the optical image of the spatial differentiation dielectric metasurface. As can be seen, the diameter of the substrate is 25.4 mm, with an effective pattern area in the center of it, whose diameter is 8 mm. The thickness of the substrate is 3 mm, and the phase gradient period is 4000 µm. The designed orientation of the nanostructure in the green box of Figure 3b is shown in Figure 3c, according to the Pancharatnam‐Berry phase design. The spatial differentiation dielectric metasurface is fabricated by the laser writing method with silicon dioxide material. The metasurface pattern is fabricated by a femtosecond pulse laser inside the glass substrate^[^ ^64^ ^]^ (50 µm below the surface of the substrate). With the high intensity of the laser irradiation, the exposed silicon dioxide (SiO_2_) substrate area will decompose into nanostructure glass (SiO_x_). By precisely modulating the illumination laser polarization, it is possible to generate nanostructures exhibiting a gradual variation in orientation. These different oriented nanostructures cause the final phase result of the metasurface.

The optical image obtained using a polariscope elucidates the detailed structure of the red circle of the metasurface, as shown in Figure 3d. The right panel is the scanning electron microscope (SEM) image, which shows the top view of the partial orange box of the metasurface. The transmittance of the metasurface in the visible spectral range (from 400 to 700 nm) is over 80%, and the average is 85% (see Note S3, Supporting Information for spectral transmittance of the spatial differentiation dielectric metasurface for broadband operation). The transfer function of the optical spatial differentiation metasurface is demonstrated as well (see Note S4, Supporting Information).

#### 2.2.2 Adaptive Liquid Prism

The structure diagram of the adaptive liquid prism is shown in Figure 3e. The adaptive liquid prism consists of two immiscible liquid materials (Type‐1 liquid and Type‐2 liquid: Water Solution and Iota Silicone Oil (705)) with different refractive indexes (1.36 and 1.57), a spacer, window glasses, a cavity, a cover, and a rotating shaft. The rotating shaft is linked to the spacer, so the spacer can be turned by turning the rotating shaft to achieve deflection of FOV. More detailed information and parameters about the adaptive liquid prism are given (see Note S5, Supporting Information).

An experimental setup is shown in Figure 3f to evaluate the FOV deflection capability of the adaptive liquid prism. A laser beam (632 nm, SuperK EXW‐6, NKT Photonics) was employed as the incident light beam. The displacement of the light spot was denoted by *s*, while *d* (65 mm) represented the distance between the adaptive liquid prism and the board. The experimental results of No.I and III light spots in Figure 3f represent maximum deflection limitations, and No.II has no deflection limitation. The deflection angle *α* is calculated by $\alpha =\arctan\left(s/d\right)$. As the results, the experiment demonstrated the effective light deflection range of the adaptive liquid prism spanning from −10.46° to 10.46°, which accords with the theoretical design.

### 2.3 Experimental Demonstration

#### 2.3.1 Field‐of‐View‐Tunable Edge Detection for Amplitude Objects via Proposed Meta‐Device

**Figure 4:**
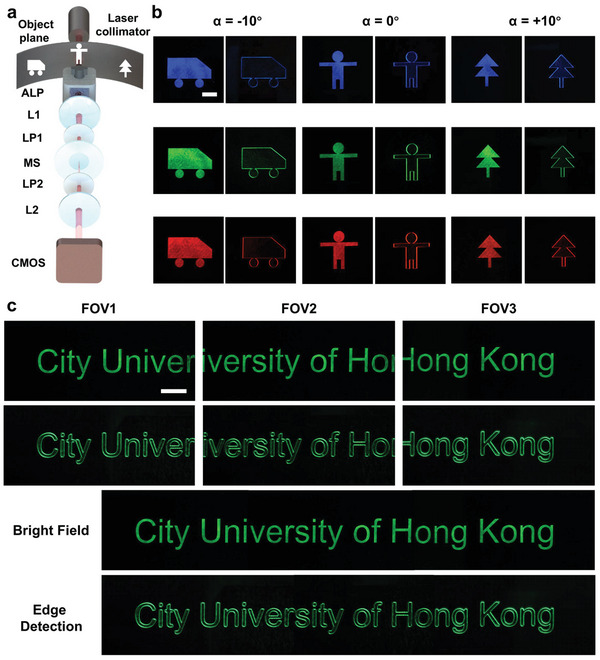
Field‐of‐view‐tunable edge detection demonstration for amplitude objects via the proposed meta‐device. a) Experiment setup. ALP: adaptive liquid prism. L1 and L2: a pair of lenses to form a 4*f* system, focal length 150 mm. LP1 and LP2: a pair of orthogonal linear polarizers. MS: metasurface. b) Bright‐field imaging (without metasurface) and edge detection (with metasurface) at 10°, 0°, and −10° field angles at wavelengths of 470, 532, and 630 nm, respectively. Scale bar: 1mm. c) Three various field‐of‐view bright‐field imaging and edge detection at wavelengths of 532 nm. And their stitching completive field‐of‐view images (below). Scale bar: 1 mm.

The experimental setup is shown in **Figure** **4**a, which is similar to Figure 2a. The setup contains a 4*f* system (L1 and L2) and a pair of orthogonal polarizers (LP1 and LP2). The spatial differentiation dielectric metasurface is placed at the Fourier plane of the 4*f* system. The adaptive liquid prism (ALP) is placed in front of the L1, and the distance between the ALP and L1 is *d* mm. The amplitude objects are located at the object plane of the 4*f* system. The distance between the amplitude objects and the ALP is *r*, and we can easily get that *r* + *d*  = *f_equ_*  (equivalent focal length of L1). Here, *r* and *d* are 100 and 50 mm, respectively. When a parallel laser is collimated from the laser collimator and illuminates the amplitude objects, the edge information can be captured by the CMOS sensor, which is placed at the back focal plane of the L2. Two types of patterns (discrete and continuous) are tested to show the performance of the proposed meta‐device. As can be seen in Figure 4b, three discrete patterns (car, people, and tree) are used as the objects to be tested, and they correspond to three different field angles in the field of view of the object space. When we detect objects at different field angles of the field of view, the laser collimator will be moved to the back of the corresponding amplitude object and illuminate this object. By adjusting the deflection angle of the adaptive liquid prism, the edge information of the object can be acquired by the CMOS sensor via the meta‐device. The proposed meta‐device can acquire the edge information of objects in different field angles. We need to mention that the spatial differentiation dielectric metasurface with different phase gradient periods has different edge imaging resolutions. This is because the spatial differentiation dielectric metasurface with different phase gradient periods split the light at different angles, which makes the radial shift different (see Notes S6 and S7, Supporting Information for detailed discussion). To demonstrate the broadband property of the meta‐device, we use three different wavelengths of a supercontinuum laser (470, 532, and 630 nm, SuperK EXW‐6, NKT Photonics) to illuminate the amplitude objects (see Note S8, Supporting Information for the power density curve of the working wavelength range of the light source). A CMOS camera (MV‐CE120‐10UC, HIKROBOT) is placed at the image plane to capture the edge image. The corresponding edge images are shown in Figure 4b, which show that the proposed meta‐device is able to work in the whole visible spectrum and has all visible spectrum responses. Broadband performance can further achieve spatial differentiation of color images. The subfield angle of the meta‐device is 4.4° can be calculated by the subfigure in Figure 4c (full original‐scale image captured by the CMOS camera), and the enhanced detected field angle is 20° because of the adaptive liquid prism, so the improved‐detected field angle by the meta‐device is ≈4.5 times the original (see Note S9, Supporting Information for detailed description about the field‐of‐view enhancement and imaging resolution of the adaptive optical spatial differentiation meta‐device).

To clarify that the proposed meta‐device can enhance the field of view, we use the same experiment setup to test. The tested amplitude objects are changed from three discrete patterns to a string of letters (“City University of Hong Kong”) with meaning that enables it to be seen as a whole. The meta‐device can capture different letters in different field angles. Finally, these subgraphs are stitched together to make a whole graph. The enhancement of the field of view is shown in Figure 4c, demonstrating that the edge information of the amplitude objects in a wider field of view can be acquired via the proposed meta‐device. Broadband demonstrations for this part are done as well. (see Note S10, Supporting Information)

#### 2.3.2 Field‐of‐View‐Tunable Edge Detection for Phase Objects via Proposed Meta‐Device

**Figure 5:**
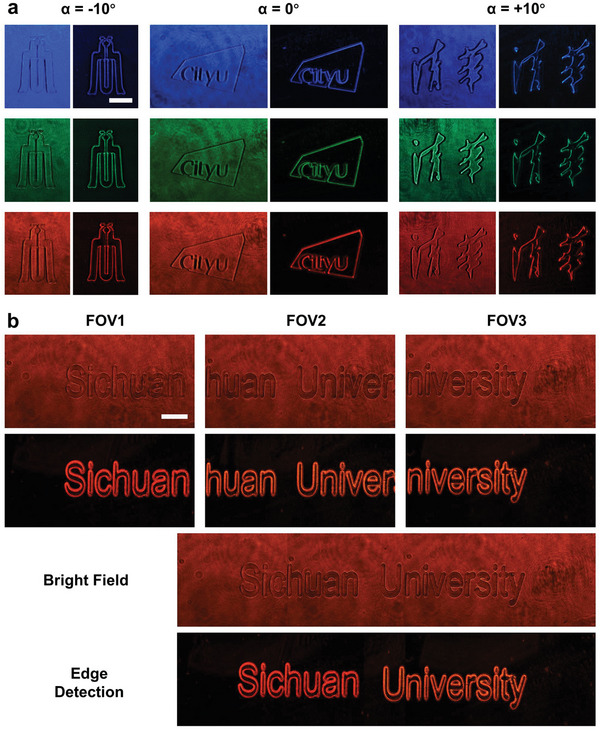
Field‐of‐view‐tunable edge detection demonstration for phase objects via the proposed meta‐device. a) Bright‐field imaging (without metasurface) and edge detection (with metasurface) at 10°, 0°, and −10° field angles at wavelengths of 470, 532, and 630 nm, respectively. Scale bar: 1 mm. b) Three various field‐of‐view bright‐field imaging and edge detection at wavelengths of 630 nm. And their stitching completive field‐of‐view images (below). Scale bar: 1 mm.

In addition to amplitude, phase is also an important feature of object information. Therefore, phase objects are also demonstrated in the following experiment. The experiment setup is the same as Figure 4a, except the tested objects changed from amplitude objects to transparent phase objects. The same experiment was done on pure‐phase objects. Three discrete patterns (the logos of three universities) are used as the objects to be tested, corresponding to three different field angles in the field of view of the object space. To showcase the broadband characteristic of the meta‐device for edge detection of phase objects, the same light sources as amplitude objects are used to illuminate phase objects. The results are shown in **Figure** **5**a. A phase object consisting of a string of letters (“Sichuan University”) is tested, as well, to demonstrate the properties of the enhancement of the field of view, as shown in Figure 5b, which clarifies the edge information of phase objects in a wider field of view can be acquired via our proposed approach. Broadband demonstrations for this part are done as well. (see Note S10, Supporting Information)

## 3 Discussion and Conclusion

The deflection angle range of the meta‐device is limited by the following: one is the rotation range for the spacer *β* of the adaptive liquid prism, which is contingent upon the structural dimensions of the adaptive liquid prism. The theoretical range of the adaptive liquid prism is from ≈−50° to ≈+50° by Equation (2). Another reason is the refractive indexes of the two immiscible liquid materials (Water Solution and Iota Silicone Oil (705)), which are 1.36 and 1.57, respectively. The greater the refractive index difference between the two liquids, the greater the deflection angle. Therefore, by further optimizing the structure and materials of the adaptive liquid prism, the deflection angle of the meta‐device can be increased, as well as reducing the size.

The proposed adaptive optical spatial differentiation meta‐device is not limited to one single wavelength because of the employment of dielectric material and the material properties of the adaptive liquid prism, which can be applied to the whole visible spectrum. Here, the broadband performance can be explained by the following: the components in the meta‐device are all visible spectrum responses, especially for the adaptive liquid prism and the spatial differentiation dielectric metasurface. The liquid materials of the adaptive liquid prism are Water Solution and Iota Silicone Oil (705), which are applicable to the entire visible light band. For the spatial differentiation dielectric metasurface, it operates based on the principle of birefringence rather than resonance. Additionally, the metasurface is constructed from SiO_2_ material, which is fabricated using pulse laser writing within a fused silica lens. The SiO_2_ material exhibits minimal dispersion, ensuring broadband operation. The multi‐frequency capabilities of the proposed meta‐device facilitate the differentiation of color images.

In conclusion, we propose an adaptive optical spatial differentiation meta‐device based on a spatial differentiation dielectric metasurface and an adaptive liquid prism, enabling broadband isotropic edge detection with field‐of‐view tunability. The proposed meta‐device breaks the limitation of the spatial information capacity, enhancing the acquisition ability of spatial information (expand to 4.5 times the original), as well as obtaining highly efficient (≈85%) isotropy edge images. We have verified the feasibility of the proposed meta‐device by experimentally performing edge detection on objects with different field angles under the visible spectrum range (from 400 to 700 nm), including amplitude objects and phase objects. The proposed meta‐device also has the advantage of easy operation, which only involves altering the deflection angle of the spacer of the adaptive liquid prism. Such a design may have further broad applications in analog image processing and provide a novel optical analog computing architecture for the next generation of wide‐angle phase contrast microscopy.

## 4 Experimental Section

### Fabrication of the Spatial Differentiation Dielectric Metasurface

The designed phase of the spatial differentiation dielectric metasurface is calculated by Equation (1). The spatial differentiation dielectric metasurface was fabricated by the laser writing method with SiO_2_. When the SiO_2_ substrate was exposed to intense laser irradiation, multiphoton ionization occurs, generating a high density of free electrons and resulting in plasma‐like behavior within the fused glass. This plasma interacts with the incoming laser beam, causing the formation of nanoscale gratings or structures, with the resulting stripes oriented perpendicularly to the beam's polarization direction. By precisely modulating the illumination laser polarization, it was possible to generate nanostructures exhibiting a gradual variation in orientation. Simultaneously, SiO₂ decomposes into SiO_x_ and O₂, with the refractive index difference between SiO₂ and SiO_x_ leading to birefringence characteristics in the long and short axes of the nanostructures. By concurrently controlling the birefringence and orientation of the nanostructures, effects analogous to those of the Pancharatnam‐Berry phase metasurface designed using electron beam lithography could be achieved.^[^ ^65^, ^66^ ^]^ These different oriented nanostructures cause the final phase result of the metasurface.

In the experiment, the femtosecond laser system utilizing mode‐locked regenerative amplification in a Yb (ytterbium‐doped potassium gadolinium tungstate) medium (Pharos, Light Conversion Ltd.), operating at a 1030 nm wavelength (photon energy ≈1.2 eV) and a repetition rate of ≈500 kHz. The laser beam was concentrated 50 µm beneath the surface of the silica sample using a spherical lens with a numerical aperture of 0.16. The pattern was fabricated by a femtosecond pulse laser inside the glass substrate (50 µm below the surface of the substrate).^[^ ^64^ ^]^ The beam polarization was adjusted through an achromatic half‐wave plate mounted on a motorized rotation stage. The sample was fixed onto a three‐axis air‐bearing translation stage system (Aerotech Ltd.), and its movement along a pre‐programmed path was managed using SCA software (Altechna Ltd.).

### Fabrication of Tested Amplitude and Phase Objects

To demonstrate the field‐of‐view‐tunable edge detection performance of the proposed meta‐device, two types of objects were fabricated to test: amplitude objects and phase objects. The commercial software AutoCAD was used to design the patterns and size of the tested objects. Mask lithography was used for amplitude object fabrication. The material of the amplitude object was SiO_2_ with a thickness of 2.3 mm, and a layer of chromium (Cr) with a thickness of ≈100 nm was over the top surface of SiO_2_ to block the light, and the transmitted part was the pattern of amplitude objects. Mask photolithography technology was used on a negative photoresist for phase object fabrication. In this process, a layer of negative photoresist (SU‐8, Microchem) with a thickness of ≈600 nm was coated over a glass (SiO_2_) substrate. A designed pattern mask was positioned between the photolithographic objective lens and the photoresist‐coated substrate to create the desired pattern. Ultraviolet light was then emitted from the light source of the lithography machine, which causes the exposed regions of the photoresist to solidify. After exposure, a development step was carried out. During development, the unexposed areas of the photoresist are selectively removed, leaving behind the desired pattern that was defined by the mask, as the phase objects. The depth between the unexposed areas and exposed areas was ≈600 nm.

## Conflict of Interest

The authors declare no conflict of interest.

## Author Contributions

L.L., J.Z., and M.K.C. conceived the idea for this work. L.L., J.Z., Z.G., and M.K.C. supervised the research. Y.Z., J.Z., and L.L. are responsible for the design and fabrication of the spatial differentiation dielectric metasurface and the adaptive liquid prism. Y.Z. and J.Z. fabricated the tested objects. Y.Z., J.Z., and J.C. build the measurement system and conduct experimental measurements. Y.G. and J.C. perform data processing and analysis. All the authors discussed the results and contributed to the preparation of the manuscript and discussions.

## Supporting information



Supporting Information

## Data Availability

The data that support the findings of this study are available from the corresponding author upon reasonable request.
