# Supplementary material for: Meta‐Device for Field‐of‐View Tunability via Adaptive Optical Spatial Differentiation
Source: Adv Sci (Weinh). 2025 Jan 13;12(9):2412794. doi: 10.1002/advs.202412794 (PMC11884584; doi:10.1002/advs.202412794)
Supplement: Supplementary file 1 — Supporting Information [file ADVS-12-2412794-s001.pdf]

ADVANCED SCIENCE
Open Access

Supporting Information

for *Adv. Sci.*, DOI 10.1002/advs.202412794

Meta-Device for Field-of-View Tunability via Adaptive Optical Spatial Differentiation

Yin Zhou, Lin Li, Junhao Zhang, Jialuo Cheng, Xiaoyuan Liu, Yunhui Gao, Zihan Geng, Lei Li*, Junxiao Zhou* and Mu Ku Chen**

Supplement Information for

Meta-Device for Field-of-View Tunability via Adaptive Optical Spatial Differentiation

Yin Zhou[1], Lin Li[2], Junhao Zhang[2], Jialuo Cheng[1], Xiaoyuan Liu[1, 3], Yunhui Gao[1], Zihan Geng[4, *], Lei Li[2, *], Junxiao Zhou[1, *], and Mu Ku Chen[1, 3, 5, *]

[1]*Department of Electrical Engineering, City University of Hong Kong, Kowloon, Hong Kong 999077, China*

[2]*School of Electronics and Information Engineering, Sichuan University, Chengdu 610065, China*

[3]*State Key Laboratory of Terahertz and Millimeter Waves, City University of Hong Kong, Kowloon, Hong Kong 999077, China*

[4]*Institute of Data and Information, Tsinghua Shenzhen International Graduate School, Tsinghua University, Shenzhen, Guangdong 518071, China.*

[5]*Centre for Biosystems, Neuroscience, and Nanotechnology, City University of Hong Kong, Kowloon, Hong Kong 999077, China*

**Zihan Geng* geng.zihan@sz.tsinghua.edu.cn

**Lei Li* leili@scu.edu.cn

**Junxiao Zhou* junxzhou@cityu.edu.hk

**Mu Ku Chen* mkchen@cityu.edu.hk

Contents:

Note 1. Comparison of previous metasurface-based optical analog computing methods and our work

Note 2. Theoretical calculation for the optical spatial differentiation metasurface

Note 3. Spectral transmittance of the spatial differentiation dielectric metasurface for broadband operation

Note 4. Transfer function demonstration of the optical spatial differentiation metasurface

Note 5. Detailed information on the adaptive liquid prism

Note 6. Characterization of the optical spatial differentiation metasurface with different phase gradient periods

Note 7. Different resolution edge imaging of the optical spatial differentiation metasurface with different phase gradient periods

Note 8. The power density curve of the working wavelength range of the light source

Note 9. The field-of-view-tunable ability description and imaging resolution of the adaptive optical spatial differentiation meta-device

Note 10. Field-of-view-tunable demonstration of a large amplitude object "City University of Hong Kong" pattern and a large phase object "Sichuan University" pattern

Supplementary Note 1: Comparison of previous metasurface-based optical analog computing methods and our adaptive optical spatial differentiation meta-device.

To compare our adaptive optical spatial differentiation meta-device with the main features of previous classic optical differentiation calculations based on metasurfaces, which can further highlight the functions and advances of the proposed meta-device. As can be seen in Table S1, compared to previous optical analog computing principles, our work has more functions, such as field-of-view tunability, broadband operation, isotropic edge detection, and both amplitude and phase object imaging.

Table S1. Comparison of previous metasurface-based optical analog computing methods and our adaptive optical spatial differentiation meta-device.

**Principle (Optical differentiation)**	**Ref.**	**FOV tunability**	**Broadband (Whole visible spectrum)**	**Isotropic**	**Both amplitude- and phase-based imaging**
Frequency-domain filtering	Ref. 1, 2, 3, 4	×	√	√	√
Spatial-domain filtering	Ref. 5, 6, 7, 8	×	×	√	×
This work		√	√	√	√

Supplementary Note 2: Theoretical calculation for the optical spatial differentiation metasurface.

The working principal diagram is shown in Figure S1. A 4*f* system consists of two lenses (L1 and L2), and the optical spatial differentiation metasurface is placed in the Fourier plane. L1 takes the object's Fourier transform, and the object's Fourier spectrum is formed at the 2*f* plane. Then, the spatial differentiation metasurface splits the Fourier spectrum light field, and the L2 takes the Fourier transform of the modified spectrum and creates the output edge information at the 4*f* plane. Here, we use the mathematical expression to explain the whole working process. The light field of the measured object at the object plane is $E_0(r_0,\theta_0)$. The L1 is used to take the object's Fourier transform, so the electric field distribution at 2*f* (Fourier plane) is $E_1(r_1,\theta_1)=\mathcal{F}\left[E_0(r_0,\theta_0)\right]$. Due to the linear polarizer 1 (LP1) (assuming x-axial direction) before the metasurface, the E_1 is x-linear-polarized, which can be expressed as $\begin{pmatrix}1 & 0\\ 0 & 0\end{pmatrix}E_1(r_1,\theta_1)$. When the electric field impacts the spatial differentiation metasurface, the electric field can be expressed as

$$E_2(r_2,\theta_2)=\left[\exp\left(i\cdot\frac{2\pi}{\Lambda}\cdot r\right)\begin{pmatrix}1 & -i\\ i & 1\end{pmatrix}+\exp\left(-i\cdot\frac{2\pi}{\Lambda}\cdot r\right)\begin{pmatrix}1 & i\\ -i & 1\end{pmatrix}\right]\begin{pmatrix}1 & 0\\ 0 & 0\end{pmatrix}E_1(r_1,\theta_1),\quad (1)$$

in which Λ presents the period of the metasurface. In this expression, $\exp\left(i\cdot\frac{2\pi}{\Lambda}\cdot r\right)\begin{pmatrix}1 & -i\\ i & 1\end{pmatrix}$ and $\exp\left(-i\cdot\frac{2\pi}{\Lambda}\cdot r\right)\begin{pmatrix}1 & i\\ -i & 1\end{pmatrix}$ represent the PB phase for LCP and RCP components, respectively. There is an orthogonal y-linear polarizer after the metasurface to get edge information, so the electric field $E_2(r_2,\theta_2)$ after the orthogonal polarizer can be given as

$$E_3(r_3,\theta_3)=\begin{pmatrix}0 & 0\\ 0 & 1\end{pmatrix}E_2(r_2,\theta_2)=$$

$$\begin{pmatrix}0 & 0\\ 0 & 1\end{pmatrix}\left[\exp\left(i\cdot\frac{2\pi}{\Lambda}\cdot r\right)\begin{pmatrix}1 & -i\\ i & 1\end{pmatrix}+\exp\left(-i\cdot\frac{2\pi}{\Lambda}\cdot r\right)\begin{pmatrix}1 & i\\ -i & 1\end{pmatrix}\right]\begin{pmatrix}1 & 0\\ 0 & 0\end{pmatrix}E_1(r_1,\theta_1),\quad (2)$$

simplify to

$$E_3(r_3,\theta_3)=\left[\exp\left(i\cdot\frac{2\pi}{\Lambda}\cdot r\right)-\exp\left(-i\cdot\frac{2\pi}{\Lambda}\cdot r\right)\right]E_1(r_1,\theta_1)=\sin\left(\frac{2\pi}{\Lambda}\cdot r\right)E_1(r_1,\theta_1). \quad (3)$$

Then, the L2 takes the Fourier transform of $E_3(r_3,\theta_3)$, so the electric field at the back focal plane of L2 can be expressed as $E_4(r_4,\theta_4)=\mathcal{F}\left[E_3(r_3,\theta_3)\right]$, and the CMOS camera at 4*f* (image plane) can obtain the light intensity, which means the output edge image. Notably, the radial shift is $\Delta=\lambda f/\Lambda$, λ is the working wavelength, and *f* is the focal distance[3]. So when different wavelengths of light are incident on the metasurface, the radial shift is different, which causes slight dispersion.

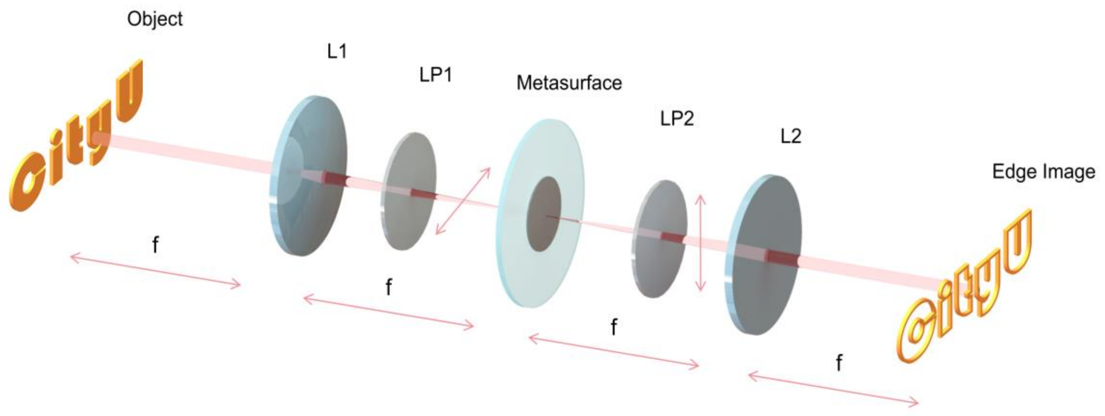

Figure S1. The working principle of the optical spatial differentiation metasurface. L1 and L2: a pair of lenses to form an optical 4*f* system. LP1 and LP2: a pair of orthogonal-linear polarizers.

Supplementary Note 3: Spectral transmittance of the spatial differentiation dielectric metasurface for broadband operation.

Figure S2 is the measured spectral transmittance of the spatial differentiation dielectric metasurface. The efficiency of the metasurface is calculated by the spectrograph. By employing a broadband laser source, the spectral transmittance with or without the dielectric metasurface is received by the spectrometer, and then the first values are divided by the second values, which is the efficiency of the dielectric metasurface under the measuring wavelengths. As can be seen, in the visible spectral range (400 nm ~ 750 nm), the transmittance is over 80%, and the average is around 85%.

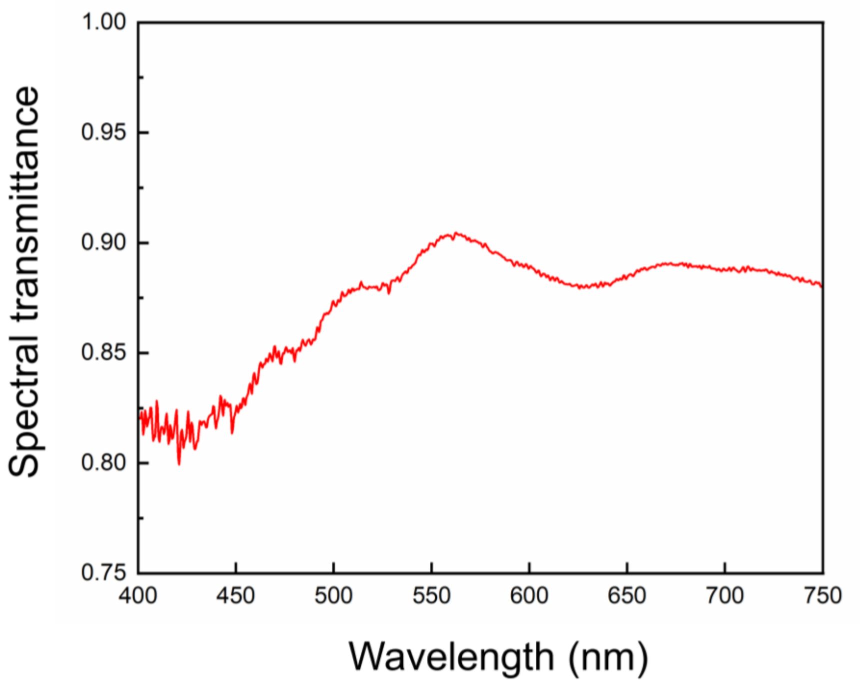

Figure S2. Spectral transmittance of the spatial differentiation dielectric metasurface in the visible spectrum range (400 nm ~ 750 nm).

Supplementary Note 4: Transfer function demonstration of the optical spatial differentiation metasurface.

We use this experiment setup to measure the transfer function of the optical spatial differentiation metasurface, which is shown in Figure S3(a). The laser beam passes through the L1 and LP1 and then impacts the dielectric metasurface, which splits into the LCP and RCP components along the radial direction. After that, the modulated laser beam passes through LP2 and L2, the function of which is to focus the two splitting beams again for the collection. Finally, the output spot is captured by a CMOS camera. The distance between the L1 and metasurface, the metasurface and the L2, and the L2 and CMOS camera are equal to the focal distance of 10 mm.

To measure the transfer function $H(k_r)$, we first measured the original laser beam spot before L1 and then calculated the electrical field distribution $E_0(x,y)=\sqrt{I_0(x,y)}$. After that, we measured the laser beam spot after L2, which means the laser beam pass through the 4*f* system with the spatial differentiation metasurface. Then, get the electric field distribution $E_1(x,y)$. The measured results I_0 and I_1 are shown in Figure S3(b). The final transfer function is calculated as $H(k_x,k_y)=\frac{E_1(u,v)}{E_0(u,v)}$, where $u=\frac{x}{\lambda f}$ and $v=\frac{y}{\lambda f}$. The transfer function result along the radial direction is shown in Figure S3(c), in which $k_r=\sqrt{k_x^2+k_y^2}$.

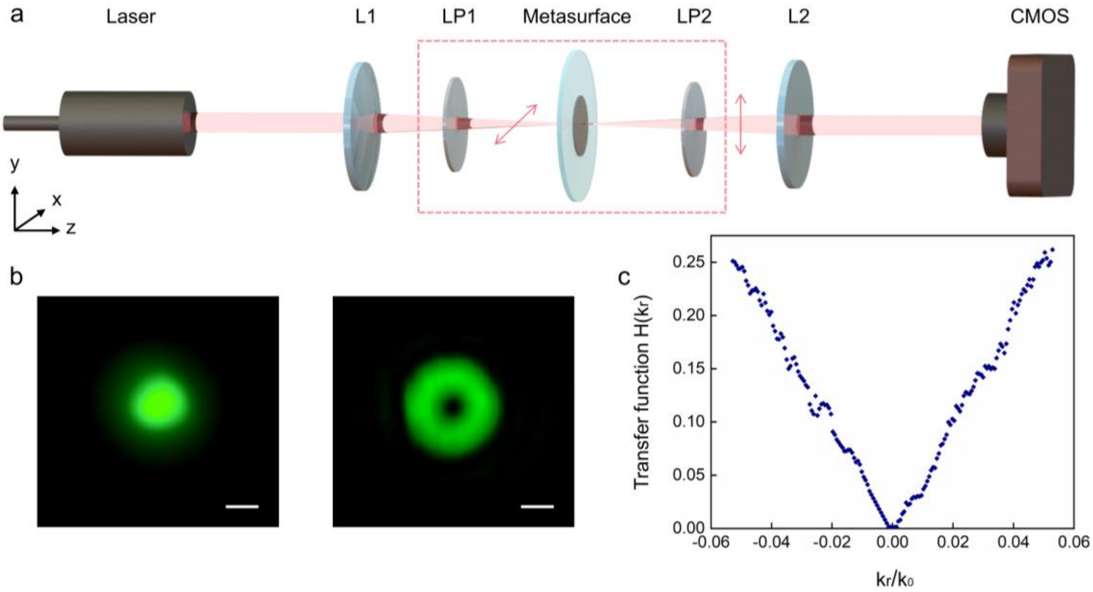

Figure S3. Measurement of the spatial transfer function of the spatial differentiation metasurface. (a) Experiment setup. L1 and L2: a pair of lenses to form a 4*f* system, focal length is 25 mm. LP1 and LP2: a pair of orthogonal linear polarizers. (b) The measurement results without (left) and with (right) the optical spatial differentiation metasurface, respectively. Scale bar: 200 μm. (c) Experimental results of the transfer function.

Supplementary Note 5: Detailed information on the adaptive liquid prism.

Here, the correlation between the angle of FOV deflection α and the rotating shaft angle of the spacer β was derived detailly based on Figure S4 below, which shows the schematic diagram of the detailed optical path of the adaptive liquid prism for field-of-view tunability. When incident light meets the first interface (from air to Type-1 liquid), the relationship between α and γ is

$$n_{air} sin(\alpha) = n_1 \sin(\gamma) \quad (1),$$

in which n_{air} is 1. Then, the light meets the second interface (from Type-1 liquid to Type-2 liquid), the relationship between β and γ is

$$n_1 sin(\gamma + 90° - \beta) = n_2 \sin(90° - \beta) \quad (2).$$

Finally, by employing trigonometric function for Eq.S(1) and Eq.S(2), the final completemathematic expression for α and β is

$$\alpha = \arcsin\left(\cos\beta \sqrt{n_2^2 - n_1^2 \cos^2\beta} - n_1 \sin\beta \cos\beta\right).$$

The components of the fabricated adaptive liquid prism are shown in Figure S5. The adaptive liquid prism consists of two immiscible liquid materials (Type-1 liquid and Type-2 liquid) with different refractive indexes, a spacer, window glasses, a cavity, a cover, and a rotating shaft. The materials of the window glass and spacer are Boron-Silicate Glass and Polymeric Methyl Methacrylate (PMMA), respectively. The liquid materials of Type-1 liquid and Type-2 liquid are the water solution and Iota Silicone Oil (705), respectively, whose densities of these two types of liquids are matched. The refractive indexes and Abbe number of these components are shown in Table S2, which shows the chromatic dispersion effects of the adaptive liquid prism. The effective physical dimension of the adaptive liquid prism is ~20 mm × ~19.9 mm.

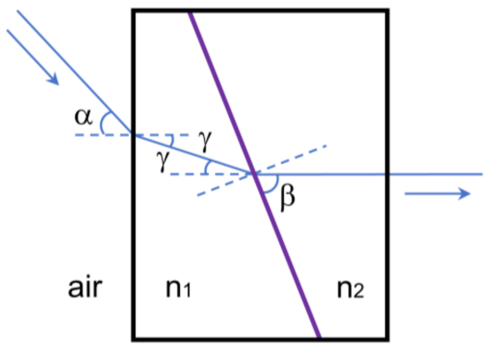

Figure S4. Schematic diagram of the detailed optical path of the adaptive liquid prism for field-of-view tunability.

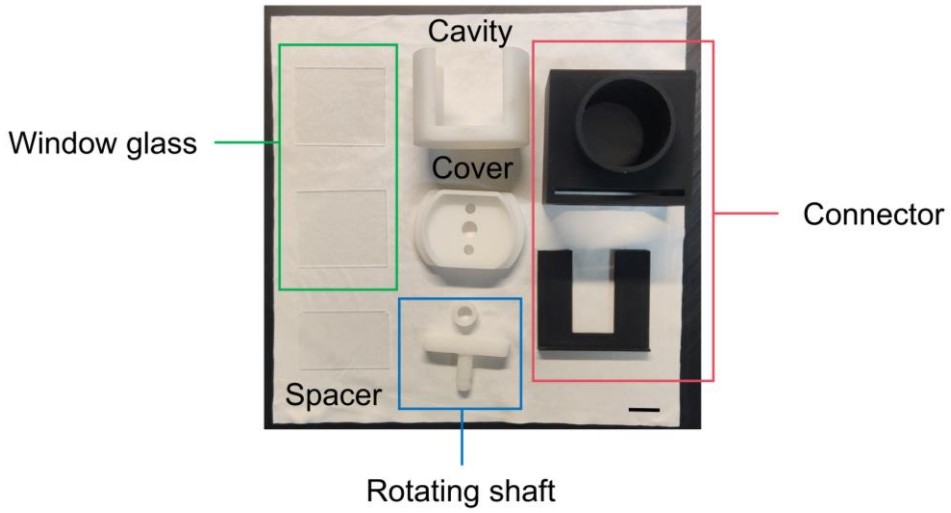

Figure S5. The components of the fabricated adaptive liquid prism. Scale bar: 1 cm.

Table S2. The refractive index and Abbe number of adaptive liquid prism materials

**Components**	**Material**	**n**	**V_d**
Window glass	Boron-Silicate Glass	1.52	65.0
Spacer	PMMA	1.49	57.4
Type-1 liquid	Water Solution	1.36	43.0
Type-2 liquid	Iota Silicone Oil (705)	1.57	34.5

Supplementary Note 6: Characterization of the optical spatial differentiation metasurface with different phase gradient periods.

The spatial differentiation dielectric metasurface with different phase gradient periods has different edge imaging resolutions. The photographs of the spatial differentiation dielectric metasurfaces with phase gradient periods Λ equal to 1000, 2000, and 4000 μm are shown in Figure S6(a-c). The diameters of the effective pattern area in the metasurface with Λ equal to 1000, 2000 μm are 6 mm, and in the metasurface with Λ equal to 4000 μm are 8 mm. Figure S6(d-f) shows the corresponding polariscope images of these spatial differentiation dielectric metasurfaces, which reflect the form-birefringent characteristics of the effective metasurface area. The zoomed polariscope images of the marked sample pattern area in Figure S6(d-f) are captured by a commercial microscope (5×), as shown in Figure S6(g-l), to show their polariscope images clearly.

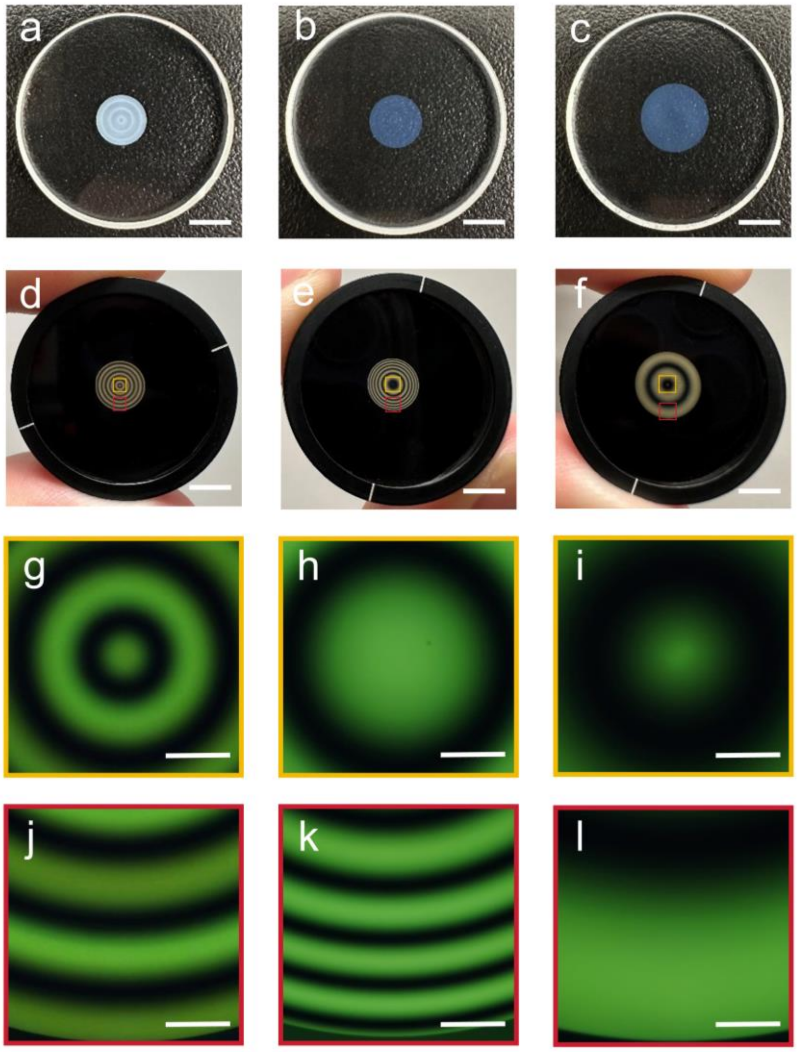

Figure S6. Optical images of spatial differentiation dielectric metasurfaces with different phase gradient periods: 1,000, 2,000, and 4,000 μm, respectively. (a-c) Without and (d-f) with a pair of cross polarizers. Scale bar: 5 mm. Polarized microscope images of (g-i) the center and (j-l) the edge of metasurfaces. Scale bar: 200 μm.

Supplementary Note 7: Different resolution edge imaging of the optical spatial differentiation metasurface with different phase gradient periods.

To demonstrate the edge imaging with different resolutions, we use the spatial differentiation dielectric metasurfaces with phase gradient periods Λ equal to 1000, 2000, and 4000 μm to detect the edge of amplitude objects of letters of "Hong Kong", as shown in Figure S7(a-c). The images without the analyzer (a pair of orthogonal polarizers) are shown in Figure S7(d-f), corresponding to metasurfaces with Λ equal to 1000, 2000, and 4000 μm. As can be seen, these images have two same images corresponding to LCP and RCP, which only have the radial-direction shift. The resolution of the edge and its sharpness can be determined by the separation between two images, as confirmed by the edge images (with a pair of orthogonal polarizers) in Figure S7(g-i).

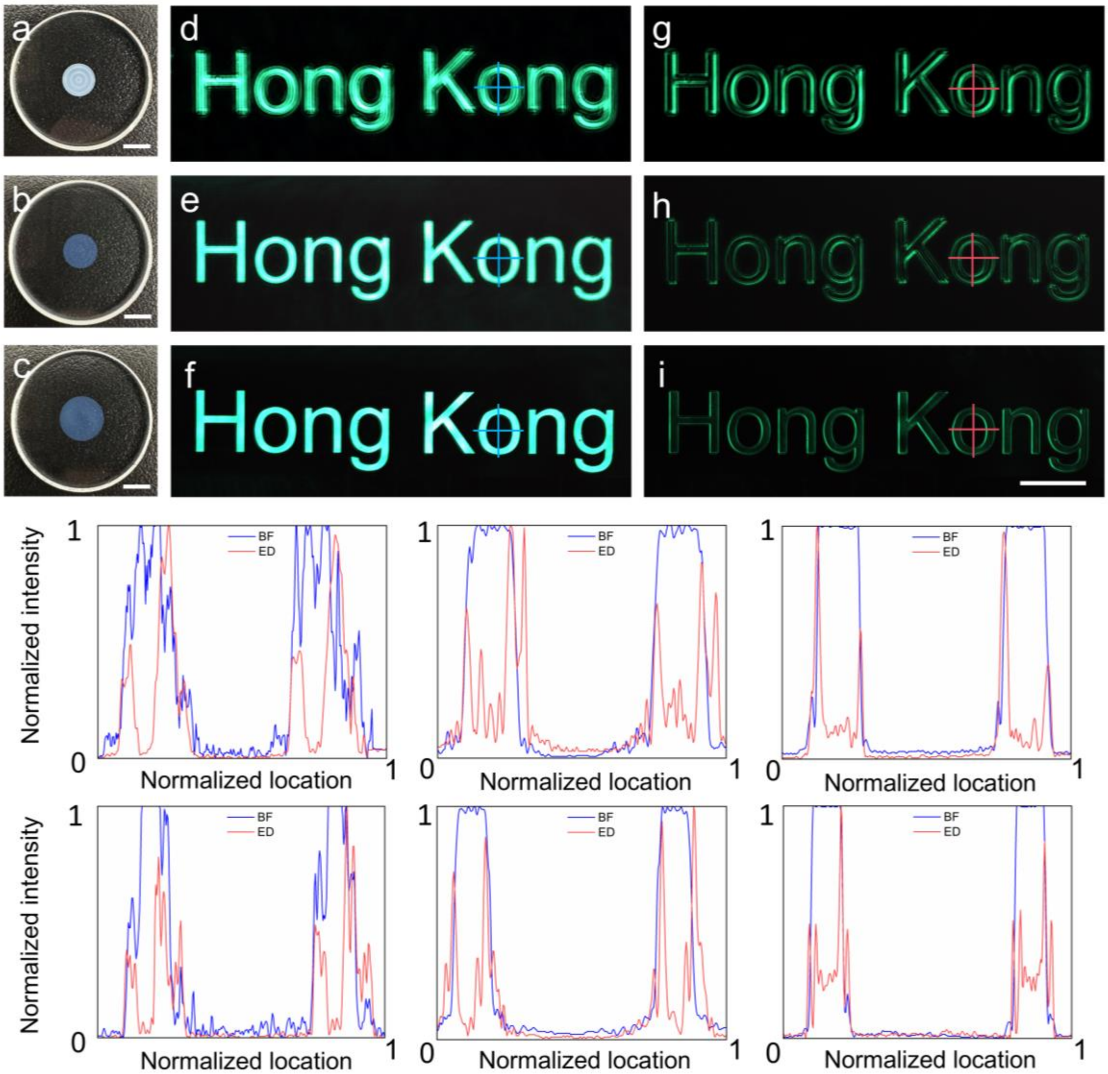

Figure S7. Edge detection at the wavelength of 532 nm with various resolutions based on the spatial differentiation dielectric metasurface with different phase gradient periods: 1,000, 2,000, and 4,000 μm, respectively. (a-c) Optical images of metasurface with different phase gradient periods: 1,000, 2,000, and 4,000 μm, respectively. Scale bar: 5 mm. (d-f) Two separated LCP and RCP images without a pair of orthogonal polarizers. (g-i) Edge images with different resolutions corresponding to different phase gradient periods. Scale bar: 1 mm. Below are the gray value graphs indicated in (d-i). The first row is the horizontal gray value of (d-f) and corresponding (g-i); the second row is the vertical gray value of (d-f) and corresponding (g-i). (BF: bright filed, ED: edge detection)

Supplementary Note 8: The power density curve of the working wavelength range of the light source.

The power density curve of the working wavelength range (400 nm ~ 800 nm) of the light source is shown in Figure S8.

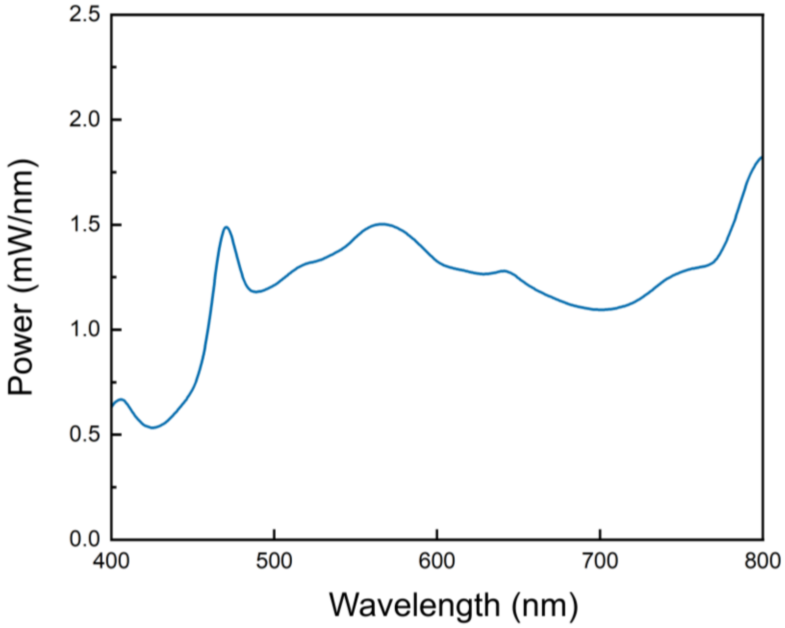

Figure S8. The power density curve of the working wavelength range of the light source.

Supplementary Note 9: The field-of-view-tunable ability description and imaging resolution of the adaptive optical spatial differentiation meta-device.

The field-of-view-tunable ability of the adaptive optical spatial differentiation meta-device is determined by the focal length of the optical 4*f* setup. The field-of-view-tunable range is 20° because of the adaptive liquid prism. When using different focal lengths of the optical 4*f* setup, as can be seen in Figure 2(a), *r* will be changed if the *d* is fixed. So the enhancement of the original field of view is different. Table S3 is the detailed data of the FOV enhancement when using different focal lengths of the 4*f* setup. Besides, the minimum size of the target object that the meta-device can detect is determined by the optical 4*f* setup, as well. When we use lenses with a large Numerical Aperture (NA) in the 4*f* system, we can detect the smaller size of the objects than when using lenses with a small NA in the 4*f* system. The NA of the lens can be calculated by $NA = n * D / 2f$, in which *n* is the refractive index, *D* is the effective aperture of the lens, and *f* is the focal length of the lens. The final imaging resolution is determined by $Resolution = 0.61\lambda / NA$. Here, for the 4*f* optical system, *D* = 50.8 mm, *f* = 150 mm, so the imaging resolution is 1.92 μm under 532 nm wavelength.

Table S3. Field-of-view enhancement for different 4*f* setup

Focal lengths of the optical 4*f* setup	Field angle	Tunable range	Enhanced detected FOV than the original
f = 100 mm	8.8°	20°	2.3 times
f = 150 mm	4.4°	20°	4.5 times
f = 200 mm	2.9°	20°	6.9 times

Supplementary Note 10: Field-of-view-tunable demonstration of a large amplitude object "City University of Hong Kong" pattern and a large phase object "Sichuan University" pattern.

By rotating the rotating shaft of the meta-device, three different field-of-view bright-field imaging (without metasurface) and edge detection (with metasurface) at wavelengths of 470 nm, 532 nm, and 630 nm can be obtained. Their completive field-of-view images in bright-field and edge detection modes can be obtained by employing image stitching technology. The experimental results of the amplitude object and phase object are shown in Figure S9 and Figure S10, respectively.

For the amplitude object "City University of Hong Kong" pattern:

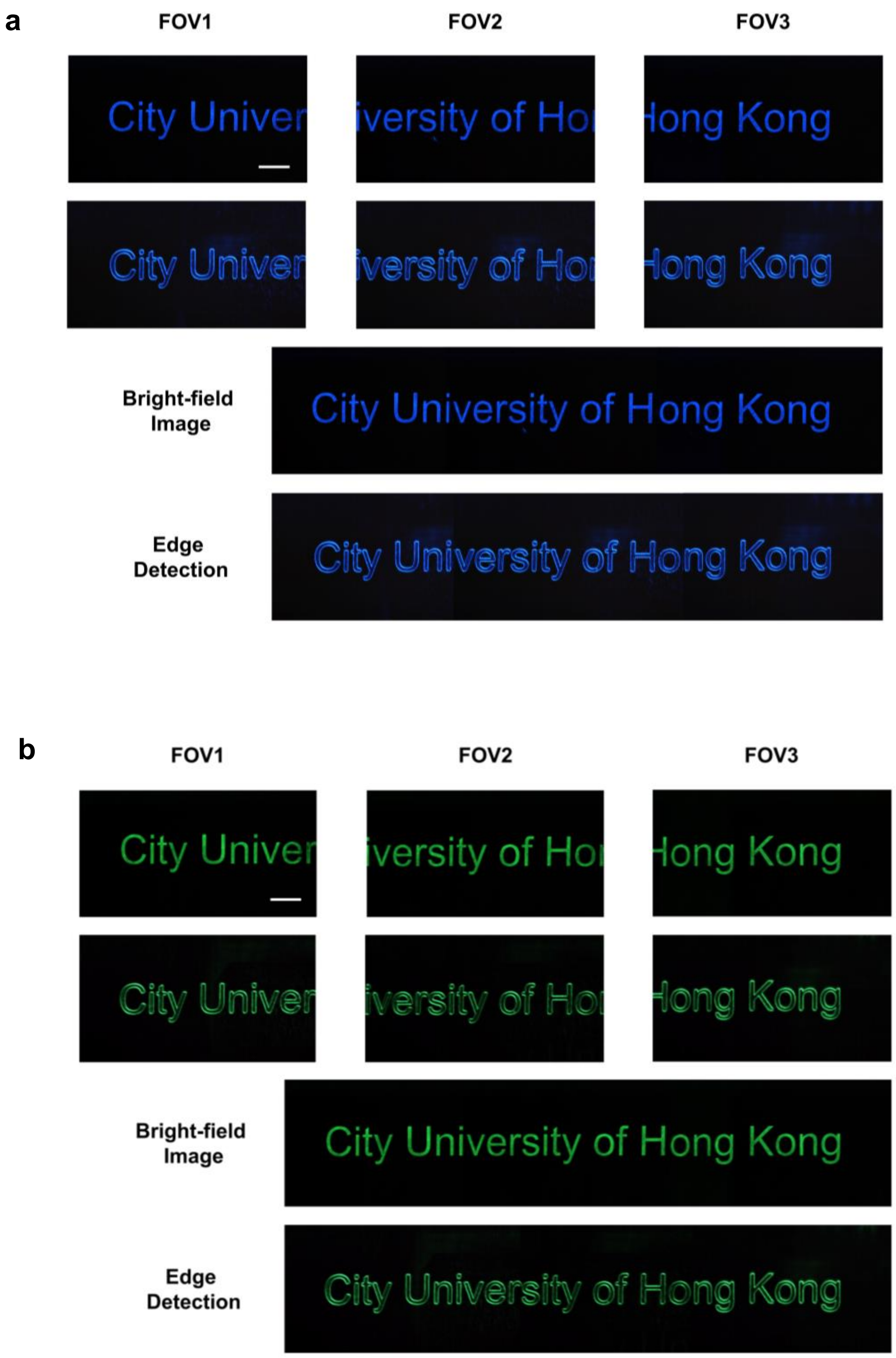

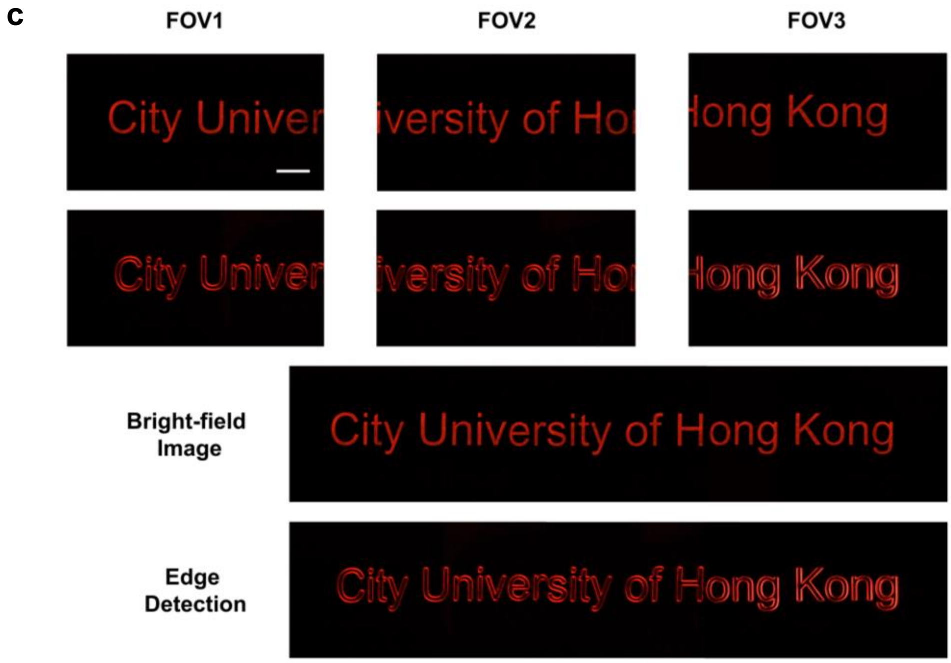

Figure S9. Three various field-of-view bright-field imaging (without metasurface) and edge detection (with metasurface) at wavelengths of (a) 470 nm, (b) 532 nm, and (c) 630 nm, respectively. And their stitching completive field-of-view images (below). Scale bar: 1mm.

For the phase object "Sichuan University" pattern:

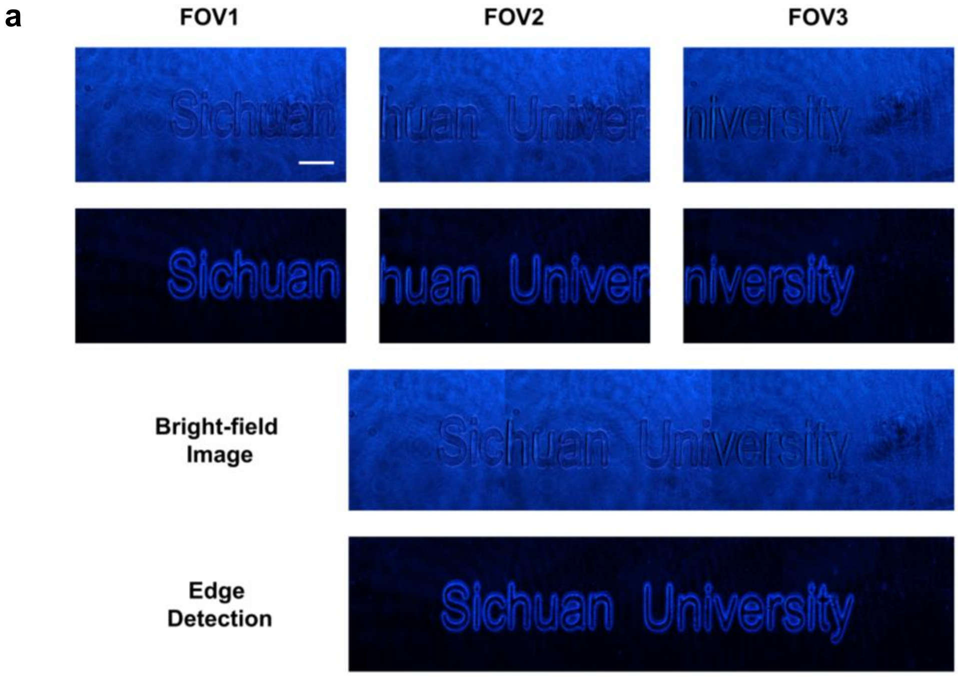

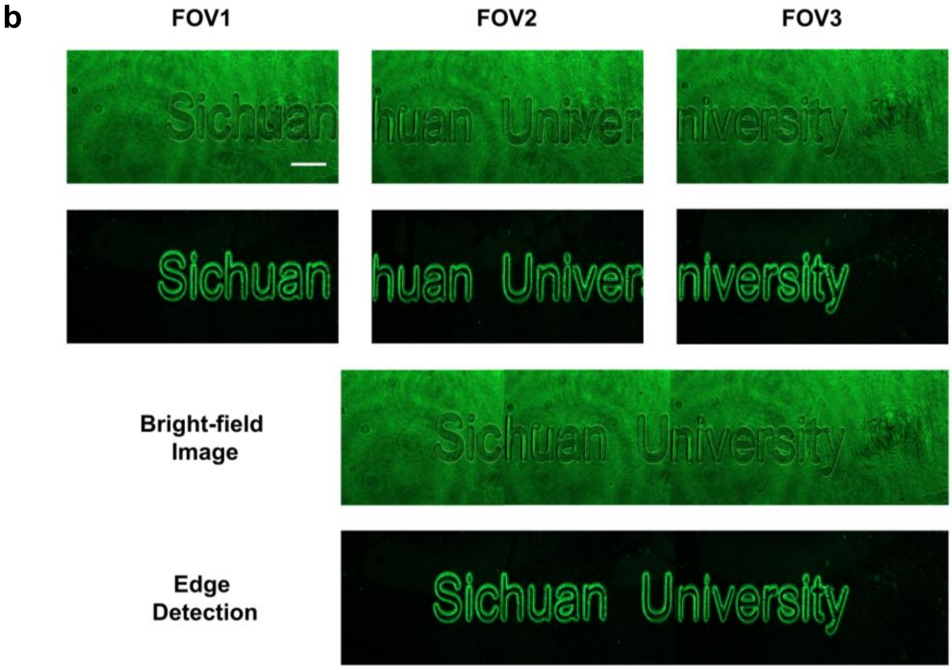

c

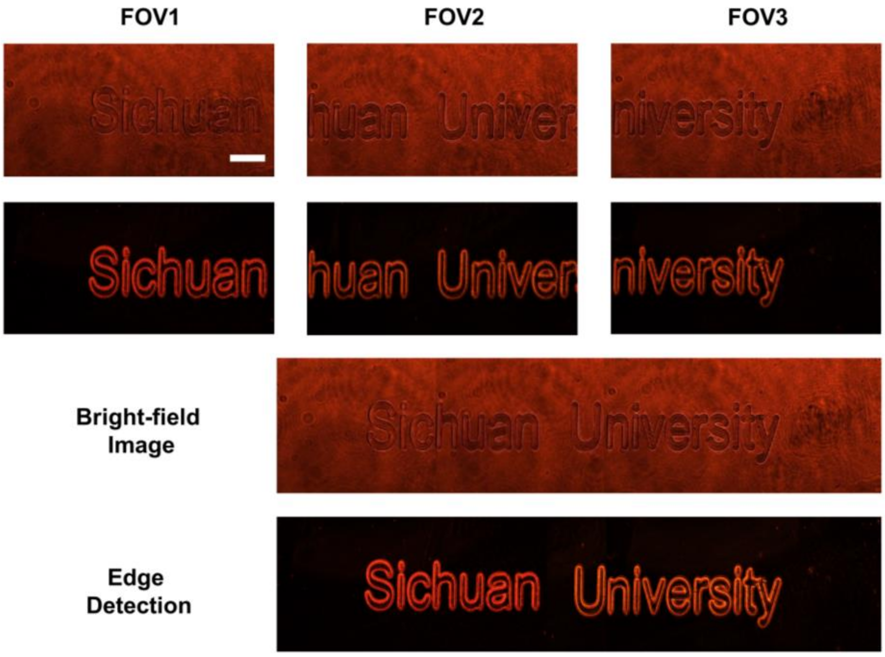

Figure S10. Three various field-of-view bright-field imaging (without metasurface) and edge detection (with metasurface) at wavelengths of (a) 470 nm, (b) 532 nm, and (c) 630 nm, respectively. And their stitching completive field-of-view images (below). Scale bar: 1mm.

Reference

1. Tanriover I, Dereshgi SA, Aydin K. Metasurface enabled broadband all optical edge detection in visible frequencies. *Nat Commun* **14**, 6484 (2023).
2. Liang X*, et al.* All-Optical Multiplexed Meta-Differentiator for Tri-Mode Surface Morphology Observation. *Adv Mater* **35**, e2301505 (2023).
3. Zhou J*, et al.* Two-dimensional optical spatial differentiation and high-contrast imaging. *Natl Sci Rev* **8**, nwaa176 (2021).
4. Zhou J*, et al.* Optical edge detection based on high-efficiency dielectric metasurface. *Proc Natl Acad Sci U S A* **116**, 11137-11140 (2019).
5. Cotrufo M*, et al.* Reconfigurable image processing metasurfaces with phase-change materials. *Nat Commun* **15**, 4483 (2024).
6. Cotrufo M, Arora A, Singh S, Alu A. Dispersion engineered metasurfaces for broadband, high-NA, high-efficiency, dual-polarization analog image processing. *Nat Commun* **14**, 7078 (2023).
7. Zhou Y, Zheng H, Kravchenko II, Valentine J. Flat optics for image differentiation. *Nature Photonics* **14**, 316-323 (2020).
8. Guo C, Xiao M, Minkov M, Shi Y, Fan S. Photonic crystal slab Laplace operator for image differentiation. *Optica* **5**, (2018).
